# Bioenergetics modelling of growth processes in parasitized Eastern Baltic cod (*Gadus morhua* L.)

**DOI:** 10.1093/conphys/coad007

**Published:** 2023-03-08

**Authors:** Marie Plambech Ryberg, Asbjørn Christensen, Christian Jørgensen, Stefan Neuenfeldt, Peter V Skov, Jane W Behrens

**Affiliations:** National Institute of Aquatic Resources, Technical University of Denmark (DTU Aqua), Kemitorvet, Building 202, Kgs. Lyngby 2800, Denmark; National Institute of Aquatic Resources, Technical University of Denmark (DTU Aqua), Kemitorvet, Building 202, Kgs. Lyngby 2800, Denmark; Department of Biological Sciences, University of Bergen, Thormøhlens Gate 53 A/B, 5006 Bergen, Norway; National Institute of Aquatic Resources, Technical University of Denmark (DTU Aqua), Kemitorvet, Building 202, Kgs. Lyngby 2800, Denmark; National Institute of Aquatic Resources, Technical University of Denmark (DTU Aqua), Willemoesvej 2, Hirtshals 9850, Denmark; National Institute of Aquatic Resources, Technical University of Denmark (DTU Aqua), Kemitorvet, Building 202, Kgs. Lyngby 2800, Denmark

## Abstract

Changes in physiological processes can reveal how individuals respond to environmental stressors. It can be difficult to link physiological responses to changes in vital rates such as growth, reproduction and survival. Here, bioenergetics modelling can aid in understanding non-intuitive outcomes from stressor combinations. Building on an established bioenergetics model, we examine the potential effects of parasite infection on growth rate and body condition. Parasites represent an overlooked biotic factor, despite their known effects on the physiology of the host organism. As a case study, we use the host–parasite system of Eastern Baltic cod (*Gadus morhua*) infected with the parasitic nematode *Contraceacum osculatum*. Eastern Baltic cod have during the past decade experienced increasing infection loads with *C. osculatum* that have been shown to lead to physiological changes. We hypothesized that infection with parasites affects cod growth negatively as previous studies reveal that the infections lead to reduced energy turnover, severe liver disease and reduced nutritional condition. To test this, we implemented new variables into the bioenergetics model representing the physiological changes in infected fish and parameterized these based on previous experimental data. We found that growth rate and body condition decreased with increased infection load. Highly infected cod reach a point of no return where their energy intake cannot maintain a surplus energy balance, which may eventually lead to induced mortality. In conclusion, parasite infections cannot be ignored when assessing drivers of fish stock dynamics.

## Teaser

Growth rate and condition of fish in relation to parasite infections were examined by bioenergetics modelling. We show that growth rate and condition decrease with increased infection load and highly infected fish reach a point of no return where energy intake is insufficient, which may eventually lead to induced mortality.

## Introduction

In marine environments, stressors can lead to changes in population dynamics of fish through their effects on vital rates, such as reproduction, growth and mortality. The dynamics and magnitude of these effects can be difficult to assess due to interactions among multiple stressors (Halpern *et al.*, 2008; Watson *et al.*, 2020). While correlations obtained from field survey data can help identifying relationships between habitat variables (physicochemical, biotic and structural) and vital rates, they provide no information on the underlying physiological processes (Horodysky *et al.*, 2015). If the underlying physiological processes behind the changes in vital rates are not understood, conclusions only based on correlations between field-based vital rate measurements and habitat variables do not allow to unequivocally identify the drivers behind population dynamics (Horodysky *et al.*, 2015). Changes in physiological processes (e.g. metabolism, enzyme activity, digestion and cardiovascular activity) can reveal how individuals respond to variations in their environment (Ricklefs and Wikelski, 2002; Young *et al.*, 2006; Horodysky *et al.*, 2015). Consequently, an understanding of how changes in physiological processes can lead to changes in vital rates is essential for our ability to interpret and predict how stressors impact the performance of animals. One way to increase our understanding is to apply bioenergetics models that quantify energy allocation under different environmental conditions, for example how warming leads to changes in energetic requirements and subsequently foraging activity and survival of fish (Holt and Jørgensen, 2015; Deslauriers *et al.*, 2017; Watson *et al.*, 2020). Furthermore, bioenergetics models may also reveal context dependent effects of stressors (Behrens *et al.*, 2018). Parasitism represents a common animal lifestyle and parasites are universal components of biological systems (Marcogliese, 2004; Kuris *et al.*, 2008; Lafferty *et al.*, 2008; Hatcher *et al.*, 2014). Parasites exploit their hosts to sustain own development and reproduction (Combes, 2001). They may affect host physiology, behaviour, reproduction or morphology (Sánchez *et al.*, 2018; Timi and Poulin, 2020), with subsequent effects on food-web stability and energy flow in both terrestrial and aquatic ecosystems (Marcogliese, 2004; Lafferty *et al.*, 2006, 2008; Kuris *et al.*, 2008; Hatcher *et al.*, 2014). Yet, infection with parasites is often neglected when describing stressors in wild fish populations (Lloret *et al.*, 2012; Timi and Poulin, 2020).

Building on a bioenergetics modelling approach used to study growth and maturation of Northeast Arctic cod (Jørgensen and Fiksen, 2006), we here assess the effects of parasite load on the energy budget and resulting growth potential of infected wild fish. The bioenergetics modelling approach basically accounts for energy intake and allocation. We use the host–parasite system between Atlantic cod in the Eastern Baltic Sea (*Gadus morhua*, hereafter referred to as cod) and the parasitic nematode *Contraceacum osculatum* as case study. This cod population is in distress, having suffered from poor growth rates and condition since the early 1990s, and today, productivity is low from a historical perspective (Eero *et al.*, 2015; Casini *et al.*, 2016b; Hüssy *et al.*, 2018; Mion *et al.*, 2020).

Currently, natural mortality of cod is estimated to be considerably higher than fishing mortality (ICES, 2020). Suggested drivers behind these trends are high fisheries pressure in the 1980s that resulted in removal of the larger fish (ICES, 2019b) and hypoxia-induced habitat compression where fish must live and forage under suboptimal environmental conditions, including low food availability (Neuenfeldt *et al.*, 2009; Plambech *et al.*, 2013; Casini *et al.*, 2016b, 2021; Neuenfeldt *et al.*, 2020). Notably, in the past decade, cod have also suffered from an increase in infections with *C. osculatum* (Haarder *et al.*, 2014; Nadolna and Podolska, 2014).

The lifecycle of *C. osculatum* involves different hosts, with grey seal (*Halichoerus grypus*) being the main final host in the Baltic Sea (Lunneryd *et al.*, 2015). The adult parasites reproduce in the stomach of the seal where the female lays her eggs, which are subsequently shed to the ambient water by egestion. Following hatching the free-living larvae are eaten by crustaceans (mainly copepods, or more rarely, gammarids; Koie and Fagerholm, 1995; Pawlak *et al.*, 2019) and/or by forage fish and who act as transport hosts (Zuo *et al.*, 2016; Nadolna-Ałtyn *et al.*, 2018). Then, these transport hosts, mainly the forage fish, are subsequently eaten by larger transport hosts, such as cod (Koie and Fagerholm, 1995). Over time, parasites accumulate in the cod liver (hence the common name “cod liver worm”), and older and larger cod typically have a higher parasite load as the nematodes never leave the liver when first encapsulated (Horbowy *et al.*, 2016; Zuo *et al.*, 2016).

The liver is a vital organ responsible for nutrient assimilation, bile production, maintenance of metabolic homeostasis and protein synthesis, and in gadoids, the liver is also the main storage site for lipid reserves (Lambert and Dutil, 1997; Hinton *et al.*, 2017). Recent studies have revealed a negative association between parasite load and nutritional condition of cod infected with *C. osculatum* (Horbowy *et al.*, 2016; Sokolova *et al.*, 2018; Ryberg *et al.*, 2020 and 2022). Infected cod also show reduced functionality of the digestive system, reduced standard metabolic rate and changes in body and plasma compositions, which together reveals that highly infected cod suffer from severe liver disease (Ryberg *et al.*, 2020). Many parasites impose an energetic cost to their host (Lester, 1971; Östlund-Nilsson *et al.*, 2005; Binning *et al.*, 2013), but in the present case, the reduced standard metabolic rate of heavily infected cod is believed to be caused by inflammations and liver tissue damage made by *C. osculatum*, leading to reduced functionality of the organ (Behrens *et al.*, accepted; Ryberg *et al.*, 2020). Impaired functionality of the liver can lead to a compromised digestive system, supported by the findings by Ryberg *et al.* (2020), revealing reduced size of the intestine and the pyloric caeca in heavily infected fish, in addition to changes in the body and liver composition, and fish energy resources in relation to the infections. These changes do not resemble a normal starvation response, and the authors therefore argued that the high infection densities, and not starvation, were the main driver of the observed changes in body composition and preferred substrate utilization of their investigated fish (Ryberg *et al.*, 2020).

Under the assumption that the energy intake decreases with increasing infection density, in the present study, we quantified how infection with *C. osculatum* may affect growth and condition of cod. We parameterized a bioenergetics model with data from recent experimental work that reveal reduced standard metabolic rate, impaired liver function and disease status of cod infected with *C. osculatum* (Ryberg *et al.*, 2020), and validated the model output against field observations obtained from scientific surveys.

## Material and Methods

We start from a previously established, state-dependent bioenergetics model (Jørgensen and Fiksen, 2006) to study the impact of parasite infections on growth in fish. The model was first developed for the Northeast Arctic stock of Atlantic cod (Jørgensen and Fiksen, 2006) to study growth and fishing-induced changes in maturation age and size. The model predicted complex life history phenomena, such as skipped spawning (Jørgensen *et al.*, 2006) at levels that were confirmed in large-scale field studies (Skjæraasen *et al.*, 2012) and has later been extended with a migration trait to predict spawning distribution (Jørgensen *et al.*, 2008*;*Opdal and Jørgensen, 2016*).* In the present study, modifications that describe infections with parasitic nematodes are added to resolve the emerging patterns in growth and condition, and the model is reparameterized for the Eastern Baltic cod. Here, we first give a brief explanation of the model with focus on what has changed when including parasites. For further details, we refer to the online supplementary material that contains a full model description. We also provide justification for the updated parameters to represent the biology of Eastern Baltic cod, while all parameters are listed in Supplementary Table S1 (supplement). This version of the model was fully recoded and implemented in Python (Harris *et al.*, 2020; Python Software Foundation, 2020), and plots were made in R (R Core Team, 2016).

### Bioenergetics model

**Figure 1:**
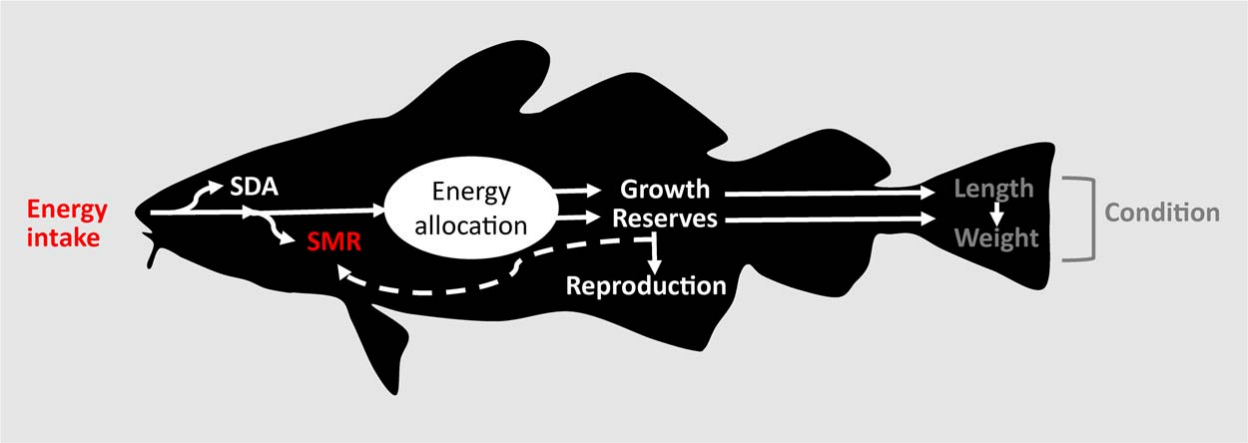
Schematic overview of the bioenergetic model examining growth and condition of fish infected with parasites. In the model the parasite causes a reduction (red colour) of maintenance cost (SMR) as well as energy intake. The standard dynamic action (SDA) is covering all energy related to processing of food for use and storage. When the energetic requirements of SMR and SDA are covered, the surplus energy is allocated toward different processes. In the present model, the fish store its energy and use it on growth and/or stored energy where the latter can be allocated to reproduction and SMR depending on the energetic status of the fish (white colour). In the case where surplus energy becomes negative (i.e. reduction in food intake), the fish can start using its stored energy for maintenance of SMR (dashed white line). Length, weight and resultant condition are states of the outcome of the energy allocation (dark grey colour).

The bioenergetics model is based on the Wisconsin framework (Hewett and Johnson, 1992), which describes how energy in the food is diverted to digestion, excretion and defecation and further cover costs of maintenance metabolism and activity (swimming) before any surplus can be used for growth and reproduction (Fig. 1).

Thus, the surplus energy is the energy intake minus the metabolic cost related to metabolic processes associated with life,


$$ surplus\ energy= energ{y}_{intake}- metaboli{c}_{cost} $$

Many of the relationships in the model depend on mass. The model differentiates between structural mass ${W}_{\mathrm{soma}}$ (g), which increases irreversibly, and the mass of the energy stores ${W}_{\mathrm{E}}$ (g), which can build up or be drawn from in times of need. The individual dies if Fulton's condition falls below a threshold value (Supplementary Table S1). Maintaining condition above its minimum thus results in storage of resources that the fish can use in periods of low food intake or for spawning. Standard metabolic rate $(\mathrm{SMR}\ \mathrm{in}\ \mathrm{kJ}/\mathrm{year}$) increases allometrically with total body mass $W=\left({W}_{\mathrm{soma}}+{W}_{\mathrm{E}}\right)$ (g),


$$ {SMR}_{\mathrm{uninfected}}={\kappa}_1\cdotp {W}^{\beta_1} $$

where ${\beta}_1\approx 0.8$ (−) is typical for fish. Net food intake $\mathrm{\phi}\ (\mathrm{kJ}/\mathrm{year}$) scales with lean body mass, as fatter fish are not necessarily better at chasing food,


$$ {\phi}_{\mathrm{uninfected}}={\kappa}_2\cdotp {W}_{\mathrm{soma}}^{\beta_2} $$

The temperature-dependent coefficients ${\kappa}_1$ and ${\kappa}_2$$(\mathrm{kJ}\cdotp {\mathrm{gram}}^{-{\mathrm{\beta}}_2}\cdotp$year^−1^) are given in Supplementary Table S1.

### Implementing infections with parasites into the model

The key additions in the present version of the model are (i) food intake-dependent parasite infection load, and (ii) recursive parasite load effects on cod food intake and energetic requirements. Focus was put on the emerging consequences of successively lowered energy intake at progressing parasite infection on cod growth and condition. Cod becomes infected following ingestion of smaller infected prey items, mainly the small planktivorous fish sprat *Sprattus sprattus*, and nematodes accumulate in the liver over time (Zuo *et al.*, 2016; Nadolna-Ałtyn *et al.*, 2018). Controlled experiments with naturally infected cod with this nematode have revealed that the physiological consequences of infection do not depend on the number of nematodes in the liver *per se*, but rather the number of nematodes per gram liver (Ryberg *et al.*, 2020). Cod is a transport host for the parasite (i.e. the parasite does not become adult and reproduce in cod; Koie and Fagerholm, 1995), so we assume in the model that new infections only arise through ingestion of infected prey. Parasite density ${\rho}_{\mathrm{parasites}}$ in the prey (parasites per kJ) is assumed constant, so the number of parasites within a cod increases during timestep *t* as


$$ {N}_{\mathrm{parasites}}\left(t+1\right)={N}_{\mathrm{parasites}}(t)+{\rho}_{\mathrm{parasites}}\ \phi \left(\mathrm{t}\right) $$

where $\phi$(*t*) is the food intake (kJ) during timestep *t*. The liver is an important energy-storing organ in cod. We described its variable weight ${W}_{\mathrm{liver}}$ as the minimal liver weight at a given ${W}_{\mathrm{soma}}$ plus additional liver tissue, depending on the amount of stored energy (see supplementary material). Infection density $I$ (parasites per g liver) at time *t* is then


$$ I(t)=\frac{N_{\mathrm{parasites}}(t)}{W_{\mathrm{liver}}(t)} $$

Experimental evidence suggests that heavily infected cod have impaired physiological and nutritional condition, particularly reduced liver function. This in turn leads to reduced energy (food) intake and reduced assimilation efficiency, resulting in less free energy available for growth and energy storage. With a greatly reduced energy budget, there is evidence that cod also reduce their SMR when infected (Ryberg *et al.*, 2020). The model incorporates these two effects on physiology from parasite infection: reduced food intake, $\phi$(*t*), mediated by slower assimilation or less successful foraging, and reduced energetic requirement, $MR(t)$, through reduction of metabolic rate:


$$ \phi (t)={\phi}_{\mathrm{uninfected}}(t)\cdotp \exp \left(\frac{-I(t)}{I_{\mathrm{intake}}}\right) $$

\begin{align*} MR(t)&={SMR}_{\mathrm{uninfected}}(t)\\&\quad\cdotp \left[{Act}_{{\mathrm{I}}_{\infty }}+\left({Act}_{\mathrm{std}}-{Act}_{{\mathrm{I}}_{\infty }}\right)\cdotp \exp \left(\frac{-I(t)}{I_{\mathrm{SMR}}}\right)\right] \end{align*}

Here ${\phi}_{\mathrm{uninfected}}(t)$ and ${SMR}_{\mathrm{uninfected}}(t)$ (both kJ/year) are food intake and standard metabolic rate of healthy fish, which are reduced in parasite-infected individuals according to the infection density $I(t)$ with a decay specific to each process (${I}_{\mathrm{intake}}$ and ${I}_{\mathrm{SMR}}$). Metabolic rate, $MR(t)$, never falls below a specific proportion ${Act}_{{\mathrm{I}}_{\infty }}$ of normal, even at the highest levels of parasite infection.

An individual is characterized by four state variables in the model: age, body length (which increases through growth), energy stored (which defines Fulton’s condition factor) and number of nematodes (acquired through ingestion of food) per gram liver weight. For every time step, these state values are updated, and the division of resources between growth and reproduction is described by the strategy parameter *u* (−). Every time step, a proportion $u$ of the free energy is allocated to storage, while the proportion $\left(1-u\right)$ is used for somatic growth and thus increases body length. For simplicity we apply fixed values *u* for immature and mature individuals, respectively, which gives a reasonable biphasic growth curve. Mortality rate is in the model based on the condition factor. Death is a stochastic process that takes into consideration an increased risk of predation and starvation, subject to individual and environmental variability, where mortality rate increases strongly below a certain threshold in body condition. Thus, this study adopts a simple, transparent criterion, namely a minimal condition K_min_, below which death is becoming more probable.

### Initialization of the model

Fish were introduced in the model at age 4 years and with a body length of 30 cm based on age–length relationships estimated by Hüssy *et al.* (2018), resulting in only adult fish in the models based on the reaction norm for Eastern Baltic cod (estimated by logistic fit of ogive) (age, length), assuming a linear (age, length) maturity reaction norm; data from ICES (2019b). A body length of 30 cm was chosen as a previous study has revealed that the proportion of cod infected with *C. osculatum* is low at this size but increases rapidly at body lengths above 30 to 35 cm (Zuo *et al.*, 2016), likely caused by a diet shift at these sizes. Initial Fulton’s condition factor was set to 0.875 for a 30-cm cod, based on Ryberg *et al.* (2020), and initial number of nematodes was set to zero. The cod in the model was followed by monthly time increments of state variables over 8 years.

### Parameterization of parasite infections

The first new parameter, ${Act}_{{\mathrm{I}}_{\infty }}$, implemented into our model reflects the reduction in standard metabolic rate of highly infected cod. Previous experimental work with infected cod (Ryberg *et al.*, 2020) showed that SMR was reduced by 23% when comparing highly infected with non-infected individuals (Supplementary Figure S1, Table 1). Using ${Act}_{{\mathrm{I}}_{\infty }}=0.77$ achieved this. The experiments also revealed that above four nematodes per gram liver weight (Supplementary Figure S1, Table 1, Ryberg *et al.*, 2020), the SMR of cod tend to be reduced, thus ${I}_{\mathrm{SMR}}=4$ (nematodes per gram liver). This indicated a non-linear dose response pattern between infection density and SMR as the cod tend to be able to cope with intermediate infection densities (i.e. ${I}_{\mathrm{SMR}}<4$) before the bioenergetics of SMR (i.e. the energetic requirements of SMR) would become more severely affected by the infections. Ryberg *et al.* (2022) revealed a strong negative relationship between nutritional condition and the infection density of cod. This was parameterized as a decrease in food intake i.e. impaired assimilation due to the infections, with ${I}_{\mathrm{intake}}$ = 5 (nematodes per gram liver) (Supplementary Figure S2, Table 1).

**Table 1:** Overview of the four variables included in the model to account for the infections with parasites in the cod

**Parameter**	**Value**	**Unit**	**Biological interpretation**
*ρ*_parasites_	8 × 10^−4^	Nematodes/kJ prey energy	Nematodes per kJ
*I*_SMR_	4	Nematodes/gram liver	Metabolic cost starts to decrease
${Act}_{{\mathrm{I}}_{\infty }}$	0.77	—	Reduction in metabolic cost
*I*_intake_	5	Nematodes/gram liver	Energy intake starts to decrease

See text for more detailed information on how each variable has been parameterized. All parameters and hence values from the original bioenergetics model for Northeast Arctic cod can be found in Supplementary Table S1.

**Figure 2:**
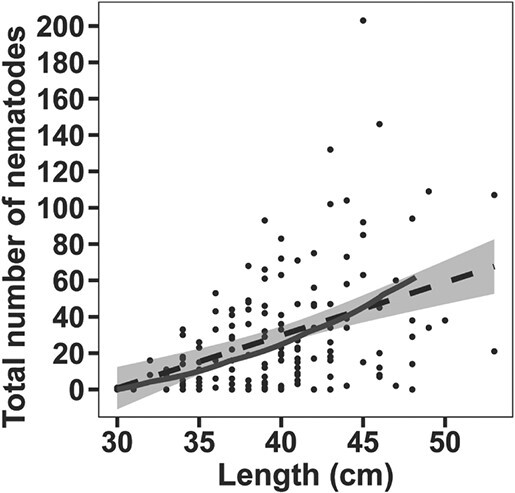
The parameterisation of *ρ*_parasites_ (nematodes/kJ prey energy) was done by comparing the observed (black line, linear regression; α = 2.95, SE = 0.58, p < 0.001) and the modelled (grey non-linear line) total number of nematodes where the model was tested with different *ρ*_parasites_ values. The observed data is derived from a previous study (Ryberg *et al.*, 2020). The resulting *ρ*_parasites_ value was 8 × 10^−4^ nematodes/kJ prey energy based on this comparison.

Due to the diverse and variable diet of cod (Neuenfeldt *et al.*, 2020), it is not trivial to estimate the parasite density in ingested food for a general cod. We started by assuming a mean energy content of sprat (5.5 kJ/g; Pedersen and Hislop, 2001), intensity of infection (i.e. number of nematodes per fish, including only infected individuals) of sprat from the Eastern Baltic Sea (i.e. 1.6 nematodes per infected fish; Zuo *et al.*, 2016) and average mean weight of an 18-cm sprat (10 gram; Casini *et al.*, 2006), which suggested that one sprat on average contains 0.03 nematodes/kJ prey energy. However, validation by setting ${\rho}_{\mathrm{parasites}}$ to this value overestimated number of nematodes as a function of cod length (Fig. 2) compared to field observations obtained from monitoring surveys. We therefore tuned this parameter value until model predictions fit observations, resulting in ${\rho}_{\mathrm{parasites}}$ = 8 × 10^−4^ (nematodes per kJ prey), which possibly reflects a limited infection success of parasites present in cod prey (Figure S3, Table 1).

### Parameterization of key parameters for the Eastern Baltic cod stock

Many of the parameters in the original model made for Northeast Arctic stock of Atlantic cod were updated to better describe the biology of the Eastern Baltic cod stock (Supplementary Table S1). In terms of energy intake, we tested the difference in the amount of surplus energy when using two different feeding levels. There have been considerable historical changes in food availability for the Eastern Baltic cod, resulting in different satiation levels in different periods. We therefore ran the model first with the historic and then with a more recent satiation level by using different values for ${\kappa}_2$. These were parameterised based on two feeding regimes identified by Neuenfeldt *et al.* (2020).

Minimum liver weight in relation to length was parameterized with data from 828 post-spawned non-infected Western Baltic cod caught between 1996 and 2018. We used data from the western stock instead of the eastern stock to avoid any bias related to infections on the estimated minimum liver weight. The minimal liver weight in relation to length was estimated by using a power law function. This resulted in an exponent of 3.17 for Western Baltic cod (ICES, 2019a), which yielded unrealistic liver weights compared to observations for Eastern Baltic cod, and an exponent of 3.4 was used instead, based on better agreement between observed and modelled liver weights (Table 1).

The relationship between the modelled weight and length from the bioenergetics model was validated by fitting a weight-at-length relationship using monitoring data from the Eastern Baltic cod stock from 2010 to 2020 (N = 828, ICES, 2019a).

## Results

### The modelled growth rate and condition

**Figure 3:**
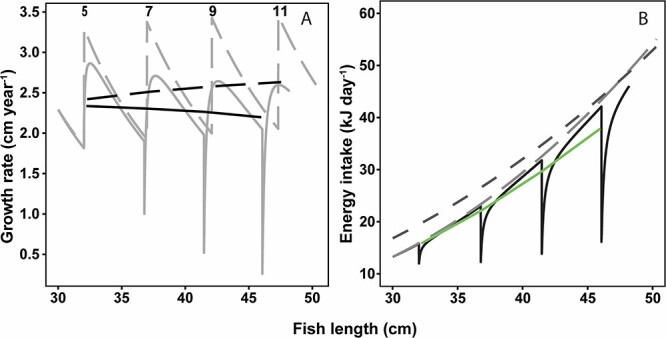
(A) Monthly resolved trajectories (grey lines) and annual trends (black lines) of growth rate in individual non-infected cod (dashed lines) at $Ac{t_{\mathrm{I}}}_{\infty }$ = 0 and infected cod (solid lines) with $Ac{t_{\mathrm{I}}}_{\infty }$> 0. Numbers above spikes represent the age (years) of the cod in the model. (B) Overall trends of daily energy intake (kJ/day) in relation to body length of infected cod. The four different lines reflect non-infected individuals with either recent satiation level (light grey dashed line) or historic satiation level (dark grey dashed line), and infected individuals with recent satiation level (black solid line) and overall trend (green solid line). The four minimum spikes in both panels A and B reflect the time after spawning where infection density (nematodes per gram liver) is very high due to the use of energy from the liver. The model output for food intake has been validated by comparison with intake levels with previous data from Neuenfeldt *et al.* (2020).

Across all sizes and ages, the modelled growth rate was lower in infected cod compared to non-infected individuals. The difference in growth rate between non-infected and infected cod was most pronounced for the largest fish, due to a more pronounced decline in growth rate of the larger, most heavily infected cod (Fig. 3A). This was the consequence of decreasing energy intake with increasing infection density in older fish (Fig. 3B). Furthermore, for fish below 45 cm, the modelled energy intake was lower at historic satiation level, compared to fish at recent satiation level (Fig. 3B). For the modelled growth rate and energy intake of infected fish, the four minimum spikes reflect the short periods directly after spawning, where infection density became very high due to the loss of liver mass resulting from energy allocated to spawning. With small livers and high densities of parasites in the liver, liver function and thus potential energy assimilation were impaired. At this point, the modelled spawning cod risked an irrevocable growth deficit.

**Figure 4:**
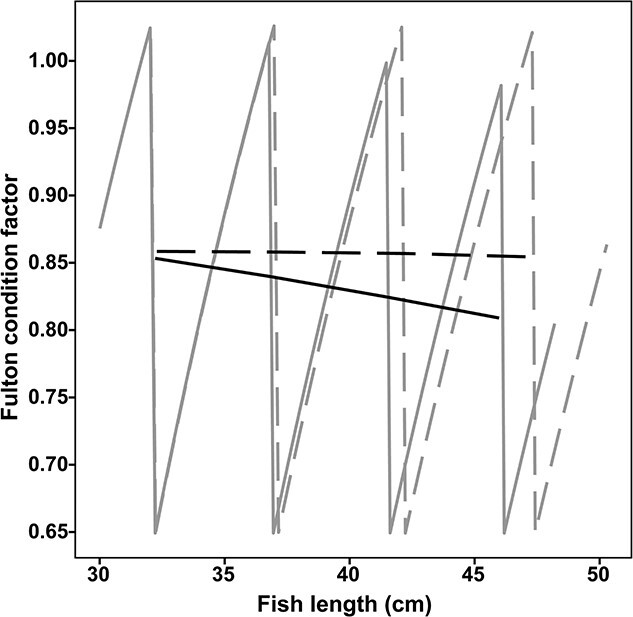
Trajectory patterns (grey lines) and overall trend (black lines) of Fulton's condition factor in individual non-infected cod (dashed lines) with $Ac{t_I}_{\infty }$(nematodes/kJ prey energy) = 0 and infected cod (solid lines) with $Ac{t_I}_{\infty }$> 0 in relation to length. Start Fulton'’s condition factor in the model was set to 0.875 (predicted condition for a 30 cm cod from a previous experimental study, Ryberg *et al.*, 2020).

Fulton's condition factor decreased with length in infected individuals but remained constant with length in non-infected individuals (Fig. 4). The recurring peaks of Fulton's condition factor reflect that cod close to maturation built liver lipid reserves in preparation for spawning (Fig. 4). More noteworthy are the time points where liver resources were depleted and infections density became very high, which caused minima for the individual trajectories for growth rate, weight-at-length and condition and for infected individuals also for energy intake. Irrespectively of the cod being infected or not, these minima only occurred on a biannual basis, suggesting that the modelled cod performed skipped spawning, thus only depleting liver reserves every second year (Figs. 3 and 4).

### The point of no return

**Figure 5:**
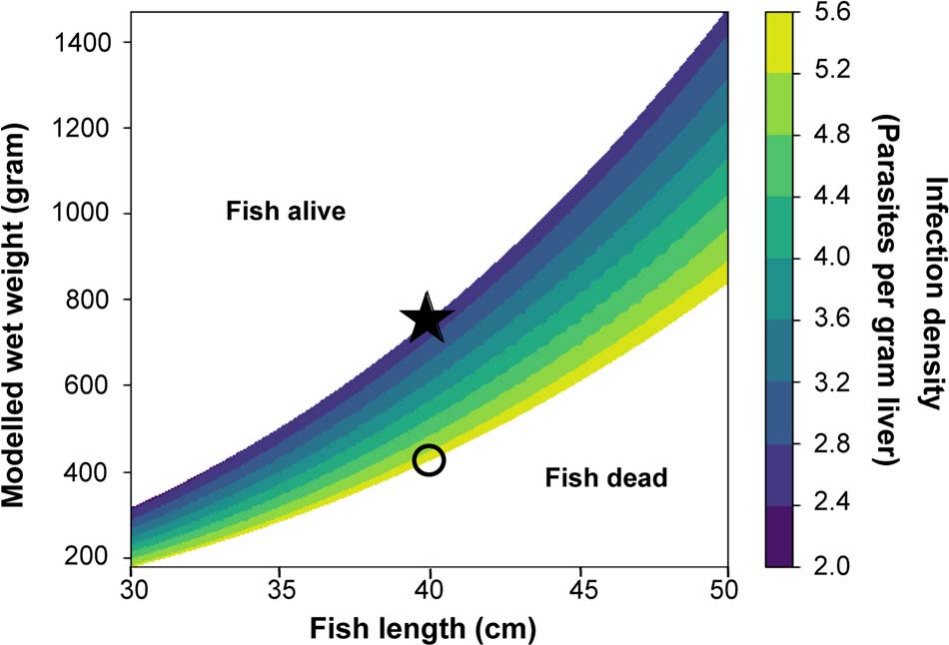
The infection level at given length and weight (contour scale) where the energy budget becomes negative in the model and the cod enters a ‘point of no return’ with respect to nutritional condition, i.e. the fish is unable to grow in length because its surplus energy balance is negative. For example, a 40-cm cod with infection density at two enters the vicious spiral when the body mass declines below 750 g (black star) and then dies when it enters below the yellow contour line in the plot (black open circle). In general, the fish is alive in the white area above and dead in the white area below the contour lines.

In the present model, the standard metabolic rate (i.e. metabolic cost) and the energy intake were gradually reduced when infection density increased, so that weakly infected cod only suffered marginally. Intake declines faster than SMR with the parameters derived for the Baltic cod stock, which means that beyond a certain infection density (defined here as the maximum infection level), the surplus energy balance became negative and the cod entered a point of no return where the condition factor continuously declined, with no possibility of reversing the trend (Fig. 5). Because liver impairment was a function of nematodes per gram liver, a cod that becomes leaner will, despite not acquiring new parasites, experience increasing infection density, deteriorating liver function and increasingly negative energy budgets until death. For example, a 40-cm cod with an infection density of two nematodes per gram liver weight entered a point of no return when the body mass declined below 750 g, illustrated by the star in Figure 5. Because metabolism is weight-dependent, lean individuals had a higher tolerance level of maximum infection at a given length and weight. Notably, however, it did not prevent a downward spiral of a cod that passed the infection density limit and simply died (open circle in in Fig. 5). The minimum infection density level of the contour scale reflects a resilience level of infection density (<2) where infection load did not result in a negative energy output.

### Comparisons with field data

**Figure 6:**
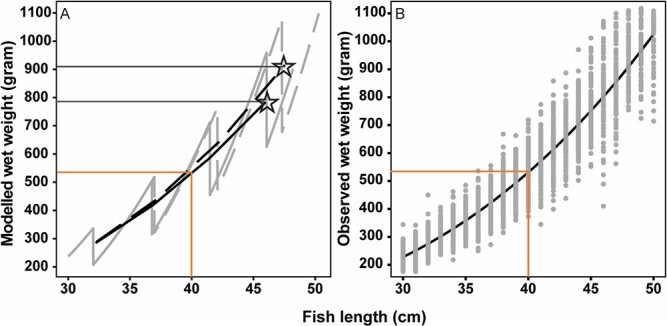
(A) Trajectory patterns (grey lines), overall trends (black lines) and final modelled weights (grey solid horizontal lines and stars) of modelled wet-weight of individual non-infected cod ($Ac{t_I}_{\infty }$=0; dashed lines) and infected cod ($Ac{t_I}_{\infty }$> 0; solid lines) in relation to fish length and (B) predictions from model fit (power law relation) of observed weight-at-length (black solid line) based on the observations (grey dots) of weight-at-length for cod between 2010 and 2020. Comparison between modelled and observed wet weight for a 40 cm cod is illustrated by the orange lines in A and B.

Modelled weight-at-length of cod compares well with field observations, both regarding the general trends (black lines) and the variations (grey lines and dots; Fig. 6A and B). Starting from approximately 40 cm of length, non-infected cod had higher weight-at-length, where an infected cod after eight years of simulation only weighed ~ 790 g compared to ~ 910 g of a non-infected conspecific (Fig. 6A).

## Discussion

Potential population-level consequences of parasite infections of individuals are often ignored by fisheries scientists and ecologists, likely because of the notorious difficulties in quantifying their influences on vital rates (Lloret *et al.*, 2012; Timi and Poulin, 2020). We show that infections with parasites potentially can reduce growth and condition of fish. This may initiate a negative spiral where a surplus energy balance is impossible to maintain, and spawning failure and increased mortality become unavoidable.

### Severity of infections

As for all higher taxa, the liver of the fish is a vital organ that carries out multiple functions, including maintaining whole body homeostasis (Rui, 2014). Based on previous experimental findings, cod with high infection loads exhibit clinical signs of a severe liver disease and impaired functionality of the organ, reflected by changes in energy composition, reduced body protein and liver lipid and increased body glycogen, in total causing a loss of energy reserves (Ryberg *et al.*, 2020). Presumably, this is due to migration of the nematodes to the liver, causing inflammatory reactions with granuloma formations in the organ, bleeding in the tissue and tunnel formations causing destruction of hepatocytes where the nematodes have moved around in the liver tissue (Behrens *et al.*, accepted; Zuo *et al.*, 2017). In support of the above experimental findings, field investigations have shown that high parasite load coincides with poor nutritional status of the cod (Horbowy *et al.*, 2016; Sokolova *et al.*, 2018; Ryberg *et al.*, 2022). In a similar host–parasite system between Atlantic cod from the southern Gulf of St. Lawrence and the parasitic nematode *Pseudoterranova decipiens*, the opposite trend has been found. Here, there seems to exist a positive relationship between the number of nematodes and the nutritional status of the fish, reflecting that the energy gain cod acquires when eating infected prey exceeds the potential negative effect of obtaining the parasite (McClelland *et al.*, 2011). It can be assumed that the energetic gain from the infected prey is the same for the Atlantic cod from the southern Gulf of St. Lawrence and the Eastern Baltic cod. However, the parasites in the two systems infect different sites of the cod and this can influence the bioenergetics differently. The main difference between *C. osculatum* and *P. decipiens* is the site of infection, as *P. decipiens* is found in the muscle tissue of cod, thereby causing less direct damage to vital organs compared to *C. osculatum*.

### Model parameterization

The weight-at-length generated by the model is consistent with monitoring data (N = 828) of weight-at-length from the Baltic Sea 2010 to 2020. The modelled energy intake fits well with previous data on feeding levels (Neuenfeldt *et al.*, 2020). The parameterization of *ρ*_parasites_ (i.e. nematodes/kJ prey energy) was adjusted to make the model fit the observations of observed total number of nematodes for specific cod lengths (Fig. 2). Moreover, the reduction in growth rate and the smaller final size in the model of infected individuals found in the present model are supported by recent findings showing that Baltic cod today have historically poor growth (Svedäng and Hornborg, 2014; Hüssy *et al.*, 2018; McQueen *et al.*, 2020). In the present model, infection density caused detrimental effects, with parameters for reduction of intake rate set to five (i.e. *I*_intake_) and SMR set to four nematodes per gram liver (*I*_SMR_), respectively. Both values are based on recent experimental data in Ryberg *et al.* (2020), where field investigations and laboratory experiments were performed to evaluate effects of *C. osculatum* on the physiological condition and health status of cod. *I*_SMR_ was set lower than the *I*_intake_ because infections with parasites first change physiological mechanisms or morphological features, which then subsequently change the performance of the infected individual (Binning *et al.*, 2017).

The assumed effects of increased infection density are supported by recent findings of decreased consumption rates (Neuenfeldt *et al.*, 2020) and condition (Casini *et al.*, 2016b, 2021) in Baltic cod in the last decades, together with findings revealing strong negative associations between parasite load and nutritional condition of Baltic cod (Horbowy *et al.*, 2016; Sokolova *et al.*, 2018; Ryberg *et al.*, 2020 and 2022). In pumpkinseed sunfish (*Lepomis gibbosus*), increased internal parasite load was also found to be associated with reduction in standard metabolic rate together with decreased escape responsiveness (Guitard *et al.*, 2022), suggesting that for this parasite–host system the parasites indirectly lead to changes in behaviour as a physiological consequence of infection. Nevertheless, deducing underlying mechanistic and physiological impacts of parasites on host bioenergetics is not trivial. Ryberg *et al.* (2020) e.g. discussed ‘the chicken or the egg’ dilemma in relation to the strong negative association between the Fulton condition factor and the increasing infection density, as animals, including fish, in poor nutritional health and with impaired immune function may be more susceptible to pathogens (Gulland, 1992; Chandra, 1997; Johansen *et al.*, 1997; Oliva-Teles, 2012). Furthermore, laboratory experiments have shown that cod in poor condition have reduced swimming performance (both endurance and sprint swimming), which in the wild may compromise their success in catching prey (Martínez *et al.*, 2003). In that case, further fasting would lead the fish to gradually use its liver reserves (Black and Love, 1986) hence resulting in an even higher infection density.

In our model, infections with *C. osculatum* first led to changes in physiological processes (i.e. reduced standard metabolic rate, impaired liver function, altered disease status; Ryberg *et al.*, 2020) and subsequently to changes in performance and behaviour. Irrespectively of causality, in the present host–parasite system the primary goal of *C. osculatum* is to reach its final host, the grey seal, so any factor that negatively affects the performance of the infected cod (including direct effects of the parasite itself) and hence aid in the trophic transfer of the parasite from the cod to the grey seal is of evolutionary benefit to the parasite.

Decreased maintenance costs with infections as found in the present study is however far from always the case in infected animals, and the cost of the parasite for the fish is often determined by the site of infection (Lafferty and Shaw, 2013), where some parasites infect sites that lead to physiological and/or behavioural changes in the host, while others choose sites as a result of nutritional preference (Lafferty and Shaw, 2013; Strømnes, 2014; Mattiucci *et al.*, 2017). Ectoparasites can induce drag to the fish resulting in increased energy demand (Östlund-Nilsson *et al.*, 2005; Binning *et al.*, 2013), or cause increased osmoregulatory burdens and costs of mobilizing immune responses (Hvas and Bui, 2022), yet others may not have any detectable bioenergetics effects on their host (Horne *et al.*, 2022). The many different bioenergetics patterns of fish infected with parasites reveal that it is not straightforward to determine whether growth and condition of fish are negatively or positively associated with a given parasitic infection.

For the present host–parasite system, it is even more complicated to predict the energetic consequences for cod in terms of preying on infected sprat, as sprat provide the cod with energy but at the same time also increase the infection load when ingested by the cod. The choice of using infection density (total number of nematodes per liver) instead of total number of nematodes to parameterize the present model is based on the argument that the liver, and the subsequent physiological responses to infection, is more likely to depend on the number of nematodes per unit of tissue mass, rather than the total number of nematodes in the liver *per se* (Ryberg *et al.*, 2020).

### From individuals to the population

Bioenergetics modelling approaches are widely used and address different aspects of physiology, ecology, aquaculture and fisheries management (Thompson and Beauchamp, 2016; Deslauriers *et al.*, 2017). In various ways, these models aid in understanding and predicting phenomena, such as malnutrition, recruitment failure or increased natural mortality rates. In our study, we found that cod had low body condition after spawning and that this was associated with a sudden high infection density due to energy allocation from the liver to gonadal build-up (Marshall *et al.*, 1999). In terms of mortality, the post-spawning period may be particularly critical for individuals, with its high infection densities. Population-level average somatic growth, recruitment and survival rates of cod vary seasonally, where critical condition of individuals after spawning is suggested to affect natural mortality (Mildenberger *et al.*, 2020). Moreover, poor body condition is known to reduce fecundity, induce skipped spawning and increase natural mortality (Dutil and Lambert, 2000; Lambert and Dutil, 2000; Rideout and Tomkiewicz, 2011; Casini *et al.*, 2016a; Mion *et al.*, 2018). Interestingly, and perhaps somewhat surprisingly, the modelled individual cod exhibited skipped spawning every second year, irrespective of being infected or not. This may reflect that cod in this population at present on average have insufficient energy intake to meet the energy requirements for spawning. Skipped spawning has indeed been observed in several cod stocks including the Eastern Baltic cod stock (Lambert and Dutil, 2000; Rideout and Tomkiewicz, 2011). In support of this, compiled information based on five decades of stomach data, Neuenfeldt *et al.* (2020) recently documented that present feeding levels are low compared to previous decades, likely a result of decreased abundance of benthic prey due to increased hypoxic areas, with no possibility of compensatory shift in diet towards more pelagic species. Skipped spawning has likewise been shown in the same bioenergetics model parameterized for Northeast Atlantic cod, where the model predicted skipped spawning to be a more regular phenomenon than previously expected in fish populations (Jørgensen *et al.*, 2006).

## Conclusion

In conclusion, building on established bioenergetics concepts, we show that a broadly applicable modelling approach can be used to complement laboratory and field investigations to unravel effects of parasites on vital rates. This contributes to the understanding of an often-overlooked driver of wild fish populations (and wild animals in general), namely parasites. Furthermore, the present modelling framework offers a platform to incorporate candidate drivers of growth and condition simultaneously, for example temperature, hypoxia and prey quantity and quality, to understand their partial and synergistic effects. With such information at hand, most likely scenarios of vital rate development can be predicted, potentially improving management measures by incorporating an element of more holistic understanding of marine ecosystems.

## Funding

This work was supported by the European Maritime and Fisheries Fund and The Danish Fisheries Agency (33113-B-17-110 and 33113-B-20-161).

## Conflicts of Interest

The authors have no conflicts to declare.

## Data Availability

The data underlying this article will be shared on reasonable request to the corresponding author.

## Author Contributions

A.C. has written the adapted model for this study and been involved in input to the analyses. C.J. has developed the original used model and provided input to the adapted model. J.W.B. participated in structuring and co-writing of manuscript. M.P.R. was responsible for the modelling and analysing part and led manuscript writing. P.V.S. participated in structuring and co-writing of manuscript; STN participated in structuring and co-writing of manuscript; all authors have been involved in the writing and edited the manuscript and approved of the final version of the manuscript.

## Supplementary material

Supplementary material is available at *Conservation Physiology* online.

## Supplementary Material

Web_Material_coad007
