# Supplementary material for: Bioenergetics modelling of growth processes in parasitized Eastern Baltic cod (Gadus morhua L.)
Source: Conserv Physiol. 2023 Mar 8;11(1):coad007. doi: 10.1093/conphys/coad007 (PMC9999110; doi:10.1093/conphys/coad007)
Supplement: Web_Material_coad007 [file web_material_coad007.zip › Web_Material_coad007.pdf]

**Supplementary Information for**

Bioenergetics modelling of growth processes in parasitised Eastern Baltic cod (*Gadus morhua* L.)

Marie Plambech Ryberg[1], Asbjørn Christensen[1], Christian Jørgensen[2], Stefan Neuenfeldt[1], Peter, V. Skov[3] and Jane W. Behrens[1,*]

[1]National Institute of Aquatic Resources, Technical University of Denmark (DTU Aqua), Kgs. Lyngby 2800, Denmark

[2]Department of Biological Sciences, University of Bergen, 5006 Bergen, Norway

[3]National Institute of Aquatic Resources, Technical University of Denmark (DTU Aqua), Hirtshals 8600, Denmark

**This document includes:**

**Supplement 1 - SUPPLEMENTARY METHODS AND REFERENCES**

**Supplement 2 – SUPPLEMENTARY TABLE S1**

**Supplement 3 - SUPPLEMENTARY FIGURES**

**Supplement 4 - SUPPLEMENTARY REFERENCES**

## Supplement 1 SUPPLEMENTARY METHODS - copied from Jørgensen and Fiksen (2006).

We use a previous established state-dependent bioenergetics model made by Jørgensen and Fiksen (2006) with specific adaptations to represent Eastern Baltic cod in the study of the energetic impact of parasite infections and subsequent effects on growth in fish.

This type of model reflects the physiological energy budget of fish and can therefore be used to study changes in energy requiring processes for example growth, condition and survival (Eq. 1):

$$(1) \qquad Freeenergy = \gamma * energy_{intake} - metabolic_{cost}$$

The free energy (kJ/time) available to somatic growth or energy storage depends on the energy intake and the food assimilation efficiency γ relative to intake (so γ = 1 - specific dynamic action - excretion fraction) minus the metabolic cost associated with life processes.

**Individual physiology**

The model includes several submodels to account for the physiology of the modelled fish. Thus, the following equations show how the physiology has been accounted for in the model by Jørgensen and Fiksen (2006). The model needs to know that body mass is divided into two compartments being soma and energy stores. The somatic weight ($W_{soma}$ (L); grams wet weight) covers irreversible structures such as the skeleton, internal organs, the neural system, and a minimum amount of muscle mass. The length-specific somatic weight (with no energy stores) is written as function of $W \sim L^{3+\varepsilon}$ as weight usually increases with length with an exponent slightly above 3 and ε for many species falls between 0.1 and 0.4 (Eq. 2):

$$(2) \qquad W_{soma}(L) = \frac{K_{min} * L^{3+\varepsilon}}{100 * L_{std}^{\varepsilon}}$$

Where $K_{min}$ is the minimum Fulton's condition factor $K = W * 100 * L^{-3}$, where weight is measured in grams wet weight and length in centimeters. If the model cannot sustain enough energy to sustain the condition above $K_{min}$, then death by starvation can be implemented in the model. In the model a certain amount of energy is defined to sustain the somatic weight at a given length.

When the fish gain weight in the model this depends on the stored energy (E) stored above the level of $W_{soma}$ (Eq. 3):

$$(3) \qquad W(L, E) = W_{soma}(L) + \frac{E}{\rho_E}$$

An average constant energy density, $\rho_E$ (kJ/gram), is defined in the model mainly reflecting the energy of proteins and lipids. This means that the model assumes that muscle proteins and lipids are stored at a constant ratio above the minimum muscle mass included in $W_{soma}$. Energy (E) has to be less than or equal to the maximum energy that can be stored ($E_{max}$) in the fish, based on physiological constraints taken from literature (Eq. 4):

$$(4) \qquad E_{max}(L) = \frac{(K_{max} - K_{min}) * \rho_E * L^{3+\varepsilon}}{100 * L_{std}^{\varepsilon}}$$

Here $K_{max}$ sets the limit for how spherical the shape of an individual fish can be and reflects the maximum Fulton's condition factor that includes $W_{soma}$ and full energy stores.

Energy expenditure is accounted for in the model as a total metabolic cost (kJ/time step). This is a product of the standard metabolic rate (SMR; i.e. maintenance costs) and a activity parameter ($Act_{std}$) that that defines the energy related to activity (Eq. 5):

$$(5) \ metaboliccost = SMR * Act_{std} = k_1 * W(L,E)^{\beta_1} * Act_{std}, Act_{std} > 1$$

Here $k_1\left(J * g^{-\beta_1} * t^{-1}\right)$ is the coefficient and $\beta_1$ is the mass exponent of the allometric function.

**Energy intake**

The energy intake (kJ/time unit) is determined by food availability in the environment together with the size of the fish as energy intake differs for given body mass or body length. Moreover, energy intake also depends on a defined satiation level (Sat) that reflects the feeding level in relation to ad libitum intake (at size). Satiation levels (at size) for Eastern Baltic cod has been resolved for a large stomach database (Neuenfelt *et al.*, 2020), both for the historic and recent ecosystem regime. In this study, parameterization for recent satiation levels were applied. As this is not naturally happening in nature a stochastic function X and seasonal cycles C(t) have been incorporated in the model to account for changes in food availability over time. Energy intake (φ) at a given weight would thus be (Eq. 6):

$$(6) \qquad \phi(W) = Sat * k_2 * W_S(L,E)^{\beta_2}$$

Where $k_2 * W_S(L,E)^{\beta_2}$ is average energy intake for a given mass.

The food assimilation efficiency $\gamma$ was also represented as an allometric relation

$$\gamma = k_3 * W(L,E)^{\beta_3}$$

Exponents and prefactors in our allometric relations for intake and metabolism are settled so that the growth speed Hansson *et al*., (1996) and Jobling (1988) from maintenance to maximum feeding levels are reproduced over the entire size range, at representative temperature (Temp) for the Baltic Sea.

## Energy allocation and growth

The bioenergetic modelling approach is built on how the free energy is allocated. Thus, a very important parameter in this context is $u$, which is defined as the core variable in the model. For every time step, a proportion *u*(age, length, stored energy ,φ) of net energy intake will be allocated to storage. With other words *u* defines the value of energy stores in the next time step (Eq. 7):

(7) $$E(t+1|u) = E(t) + u(\phi - MR)\delta_{store}, E \leq E_{max}$$

The variable $\delta_{store}$ is the assimilation efficiency for the conversion of ingested energy to stores. This value is commonly higher than the assimilation efficiency for growth of somatic structures ($\delta_{growth}$) as $\delta_{store}$ includes the relationship between stored energy and spawned eggs embody energy losses. Thus, when *u* is defined in the model the proportion (1-*u*) is allocated to somatic growth to a new length in the next time step $L(t+1 \vee u)(Eq.8)$:

(8) $$L(t+1|u) = \left[L(t)^{3+\varepsilon} + \frac{(1-u)(\phi - MR) * \delta_{growth} * 100 * L_{std}^{\varepsilon}}{K_{min} * \rho_s}\right]^{\frac{1}{3+\varepsilon}},$$

$$L(t+1) - L(t) \leq \triangle L_{max}$$

This equation states that growth is allometric with the exponent (3+ε), new tissue added in the next time step depends on the availability of food, assimilation efficiency, and the energy density of somatic tissue ($\rho_s; \frac{J}{gram}$).

## Reproduction and migration

Both reproduction and migration are two energetic processes that requires energy. The duration of these events is therefore incorporated in the model both as an energetic cost of migration and as a proportion of energy directed to spawning. The time required for the migration $T_m$ (t) is the migration distance $D_m$ (m) divided by the swimming speed (Eq. 9):

(9) $$T_M = \frac{D_M}{(U_S + U_C)}$$

Where $U_S\left(\frac{m}{timestep}\right)$ is the average or typical swimming speed during migration and $U_C\left(\frac{m}{timestep}\right)$ is the speed of possible currents that must be taken into consideration. Thus, the energetic cost of migration, $E_M(J)$ can be written (Eq. 10):

(10) $$E_M(W,L) = SMR(W)\left[\left(\frac{k_4 * U_S^{1.5}}{L} + 1\right) - Act_{std}\right] * T_M, E_M \geq 0$$

The expression $k_4 * U_S^{1.5} * L^{-1} + 1$determines an activity parameter like $Act_{std}$ from swimming speed and body size. For more details about different migration conditions please see the model description in Jørgensen and Fiksen (2006).

## Supplement 2 - SUPPLEMENTARY table S1

All variables included in the model (except the variables related to the infections) are found in table S1 below.

***Table S1.*** *All parameters included in the model are shown in this table. Both the original variables from the original bioenergetics model for Northeast Arctic cod (Jørgensen and Fiksen, 2006) that were not changed in the present study, and variables that were change in the present bioenergetic model to meet the biological information of Eastern Baltic cod.*

| **Paramete r** | **Value** | **Unit** | **Biological interpretation** | **Reference** |
|---|---|---|---|---|
| **ε** | 0.03879 | | Value of coefficient above 3 for allometric scaling between body mass and length | ICES, 2019a |
| **$K_{min}$** | 0.65 | $g/cm^3$ | Minimum condition factor | Casini et al., 2016a |
| **$K_{max}$** | 1.22 | $g/cm^3$ | Maximum condition factor | Own estimate |
| **$D_m$** | 40 | km | Distance for spawning migration | Own estimate |
| **$U_{current}$** | 0.0 | cm/year | Current during spawning migration | Own estimate |
| **$Mat_{reac}$** | 1.3754*age + 0.1194*length - 8.146 = 0 | | Reaction norm logistic fit of ogive ~ (age,length) from BITS data, assuming a linear (age,length) maturity | ICES, 2019a |
| **$Spawn_{min}$** | 50 | % | Percentage of energy store for spawning | Own estimate |
| **$F_{feeder}$** | 0.4 | $Year^{-1}$ | Fisheries mortality | Margit Eero, pers. comm. 2018 |

| | | | | |
|---|---|---|---|---|
| **$F_{spawner}$** | 0.4 | $Year^{-1}$ | Fisheries mortality | Margit Eero, pers. comm. 2018 |
| **$M_{natural}$** | $0.5*(length/20)^{-0.75}$ | $Year^{-1}$ | Natural mortality | Margit Eero, pers. comm. 2018 |
| **$M_{spawn}$** | $0.1*(length/20)^{-0.75}$ | $Year^{-1}$ | Increased mortality during spawning and migration | Margit Eero, pers. comm. 2018 |
| **Temp** | 7.0 | ˚C | Baltic reference temperature | Margit Eero, pers. comm. 2018 |
| **$Sat_{historic}$** | $0.40 - 0.1\left(1.0 - exp\left(\frac{length-20.0}{30}\right)\right)$ | | Historic satiation level | Neuenfeldt *et al.*, 2020 |
| **$Sat_{recent}$** | $0.25 - 0.15 * \left(1.0 - exp\left(\frac{length-20.0}{30}\right)\right)$ | | Recent satiation level | Neuenfeldt *et al.*, 2020 |
| **$H_0(L)$** | $10^{-4.64}*length^{3.4}$ | gram | Minimal liver weight | Own estimate |
| $\rho_{parasites}$ | $8*10^{-4}$ | nematodes/ kJ prey energy | Nematodes per kJ | Own estimate |

**Parameters used from Jørgensen and Fiksen (2006)**

| | | | | |
|---|---|---|---|---|
| $L_{std}$ | 70 | cm | Length for which Kmin and Kmax are defined | |
| $\rho_E$ | 8700 | J·g–1 | Energy density of muscle and liver energy stores | |
| $\rho_S$ | 4000 | J·g–1 | Energy density of somatic tissue | |

| | | | |
|---|---|---|---|
| $Act_{std}$ | 1.25 | - | Proportional increase in metabolic rate as a result of activity |
| $\kappa_1$ | 22294 | J·g–β1·year–1 | Coefficient of allometric metabolic function |
| $\beta_1$ | 0.828 | - | Exponent of allometric metabolic function |
| $\kappa_2$ | 22986 | J·g–β2·year–1 | Coefficient of allometric feeding function |
| $\beta_2$ | 0.802 | | Exponent of allometric feeding function |
| $k_3$ | 1.252 | g–β3 | Coefficient of allometric food assimilation efficiency |
| $\beta_3$ | -0.051 | | Exponent of allometric food assimilation efficiency |
| $\kappa_4$ | $5.7\ 10^{-10}$ | | [year^1.5 / km^0.5] Coefficient for empirical cost of swimming function |
| $\delta_{store}$ | 0.4 | | Efficiency of storing ingested |

## Supplement 3 - SUPPLEMENTARY FIGURES

### *Figure S1: Parametrisation of $I_{SMR}$ and the $Act_{I\infty}$*

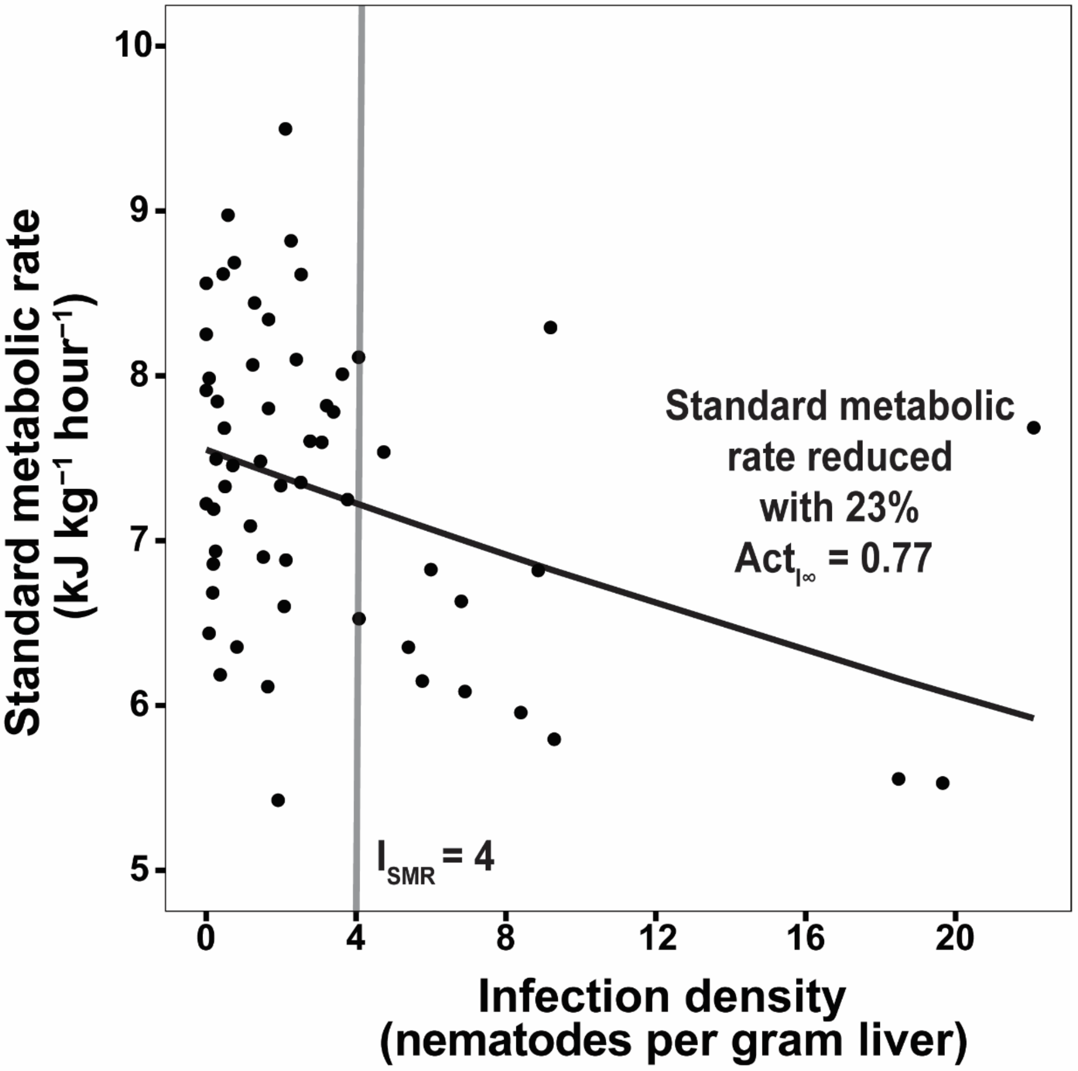


**Figure S1.** The standard metabolic rate in relation to different levels of infection densities. Data points represent respirometry measurements from individual cod and the black solid line illustrates the model fit of a previous analysis on this data (Ryberg *et al*., 2020). In the present model, this data has been used to parametrize the $I_{SMR}$ (grey vertical line) and the $Act_{I_\infty}$. The $I_{SMR}$ is defined to four nematodes/gram liver, indicating effects from the infections on the maintenance cost above this infection density level. Redrawn after Ryberg *et al*., 2020.

***Figure S2: Parametrisation of $I_{intake}$***

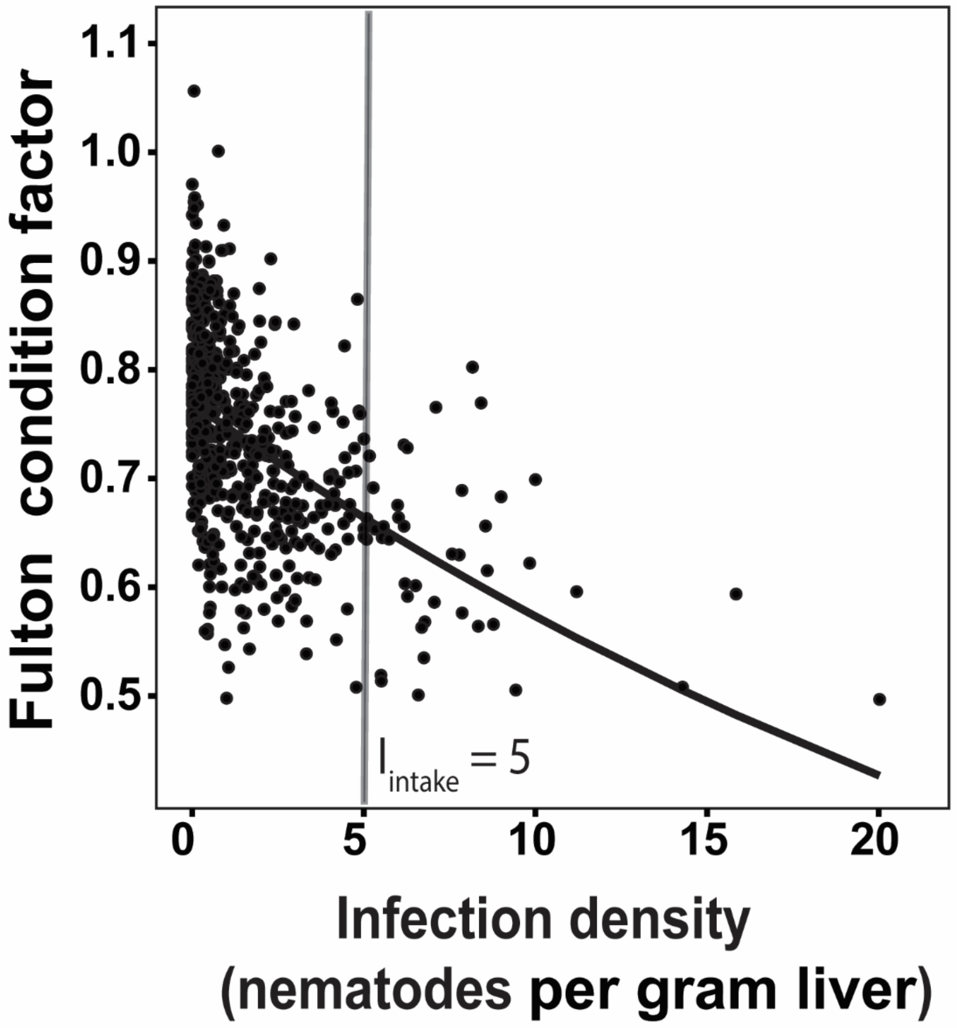


**Figure S2.** Fulton's condition factor in relation to infection density. Data represent individual cod and the black solid line illustrates the model fit of a previous analysis on this association (Ryberg *et al.*, 2022). In the present model, this data has been used to parametrize the $I_{intake}$ (grey vertical line) defined to five nematodes/gram liver, indicating reduction in the energy intake above this infection density level. Redrawn after Ryberg *et al.*, 2022).

***Figure S3: Sensitivity analysis of $\rho_{parasites}$ (nematodes/kJ)***

The model is very sensitive to $\rho_{\text{parasites}}$ (nematodes/kJ prey energy) in terms of number of nematodes for given lengths. The figure below (Figure S3) shows model outputs of total number of nematodes in relation to length with different $\rho_{\text{parasites}}$ values (factor(type)). The $\rho_{\text{parasites}}$ value with that best reflect the observed number of nematodes in the cod livers was found to be $8*10^{-4}$ (light brown line in Figure S3).

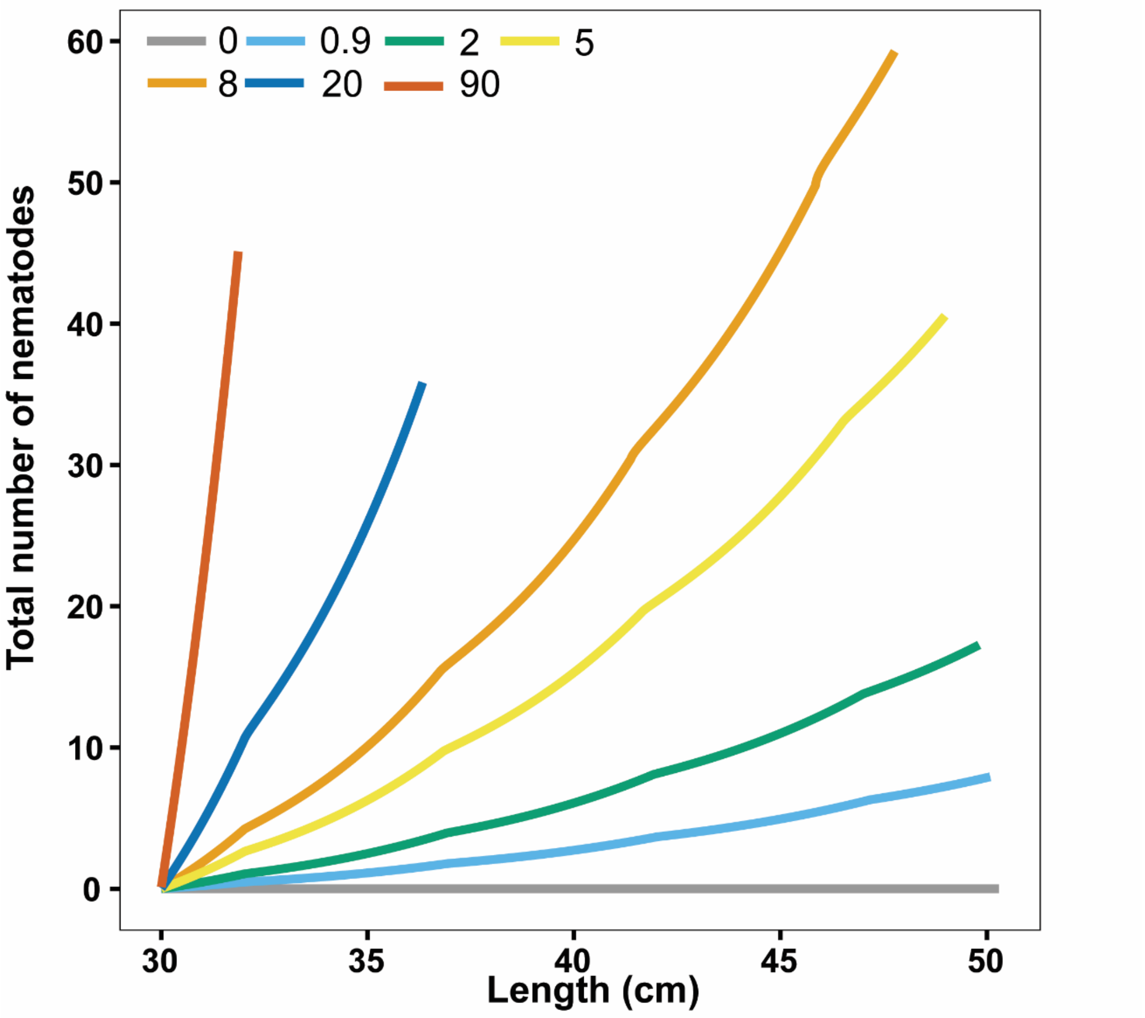


***Figure S3.*** *Relation between total number of nematodes and length for seven different values of $\rho_{parasites}$ (nematodes/kJ prey energy) used in the model. The $\rho_{parasites}$ values ($10^{-4}$) are shown in the figure and represented by seven different colours. The light brown line and $\rho_{parasites}$ value 8 is used in the model.*

## Supplement 4 - SUPPLEMENTARY REFERENCES


Casini M, Eero M, Carlshamre S, Lövgren J (2016a) Using alternative biological information in stock assessment: condition-corrected natural mortality of Eastern Baltic cod. *ICES Journal of Marine Science*, *73*: fsw117.

Hansson S, Rudstam LG, Kitchell JF, Hilden M, Johnson BL, Peppard PE (1996) Predation rates by North Sea cod (Gadus morhua) — predictions from models on gastric evacuation and bioenergetics. *ICES J. Mar. Sci. 53*: 107–114.

ICES (2019a) *Baltic International Trawl Survey*. http://ecosystemdata.ices.dk

Jobling M (1988) A review of the physiological and nutritional energetics of cod, Gadus morhua L, with particular reference to growth under farmed conditions. *Aquaculture, 70*: 1–19.

Jørgensen C, Fiksen Ø (2006) State-dependent energy allocation in cod (Gadus morhua). *Canadian Journal of Fisheries and Aquatic Sciences*, 63: 186–199.

Neuenfeldt S, Bartolino V, Orio A, Andersen KH, Ustups D, Kulatska N, Andersen NG, Niiranen S, Bergstro U, Casini M (2020) Feeding and growth of Atlantic cod (Gadus morhua L.) in the eastern Baltic Sea under environmental change. *ICES Journal of Marine Science*, 77: 624–632.

Ryberg MP, Huwer B, Nielsen A, Dierking J, Buchmann K, Sokolova M, Krumme U, Behrens JW (2022) Parasite load of Atlantic cod Gadus morhus in the Baltic Sea assessed by the liver category method, and associations with infection density and critical condition. *Fisheries Management and Ecology*, 29: 88-99.

Ryberg MP, Skov PV, Vendramin N, Buchmann K, Nielsen A, Behrens JW (2020) Physiological condition of Eastern Baltic cod, Gadus morhua, infected with the parasitic nematode Contracaecum osculatum. *Conservation Physiology*, 8: coaa093.
